# Synthesis and Biological Assessment of 2-Hydroxyiminoethanones as Anti-Inflammatory and β-Amyloid Aggregation Inhibitors

**DOI:** 10.22037/ijpr.2019.15567.13181

**Published:** 2019

**Authors:** Mehdi Valipour, Issa Davaji, Niusha Abedi, Mahsa Rajabi, Tuba Tüylü Küçükkılınç, Beyza Ayazgök, Hamid Irannejad

**Affiliations:** a *Department of Medicinal Chemistry, Faculty of Pharmacy, Mazandaran University of Medical Sciences, Sari, Iran. *; b *Student Research Committee, Mazandaran University of Medical Sciences, Sari, Iran.*; c *Department of Pharmacology & Toxicology, School of Pharmacy, Shahid Beheshti University of Medical Sciences, Tehran, Iran. *; d *Department of Biochemistry, Faculty of Pharmacy, Hacettepe University, Sihhiye, 06100 Ankara, Turkey.*

**Keywords:** Synthesis, Anti- inflammatory, Alzheimer′s disease, hydroxyiminoethanone, β-amyloid aggregation

## Abstract

Alzheimer’s disease (AD) is a neuroinflammatory based pathologic state in which β-amyloid aggregates are major devastating agents. In this study, a series of 2-hydroxyiminoethanones were synthesized and evaluated as anti-inflammatory in carrageenan and formalin tests and inhibitors of β-amyloid aggregation. Compounds **1-10b** were synthesized through a two-step reaction. Results: Compounds **1-5b** showed more β-amyloid disaggregation ability than reference drugs rifampicin and donepezil and compound **2b** was the best compound in this series and could reduce the extent of amyloid aggregation to 50.9%. Interestingly, compounds **1b** and **3b** showed significant anti-inflammatory activity in carrageenan-induced paw edema compared to control group and equivalent to the reference drug indomethacin. 2-Hydeoxyiminoethanones are privileged scaffold for further drug research and development as anti-neuroinflammatory and neuroprotective agents.

## Introduction

In body′s normal reactions, inflammation is a protective process, but excessive inflammatory response can cause tissue damages Tracey (1). In numerous studies, it has been identified that inflammation is an important factor in a large number of diseases such as atherosclerosis (2), rheumatoid arthritis (3), diabetes (4), multiple sclerosis (5), and also neurodegenerative disorders (6). Also, scientific findings indicate that neurodegeneration is associated with the presence of inflammation in a variety of neurodegenerative and neurological diseases, especially in Alzheimer′s disease (7). This matter is proved by increased levels of pro-inflammatory cytokines, such as IL-6 or TNF-α (8). Inflammatory response in the CNS is called neuro-inflammation. In the neuro-inflammation process, astrocytes and microglial cells are the main cells which are involved (9). In addition, Prostaglandins are involved in the modulation of neuro-inflammation and are produced by the cyclooxygenase (COX) enzymes. It is known that COX-1 and COX-2 are normally expressed in the brain, and their involvement in neurodegeneration has been proved in numerous studies (10-12). Scientific studies have documented a reduced prevalence of Alzheimer′s disease among users of Nonsteroidal anti-inflammatory drug (NSAIDs) (13).

Based on scientific studies, extracellular amyloid β (Aβ) plaques and intra neuronal deposits of neurofibrillary tangles (NFTs) are responsible for the progression and development of AD (14, 15). β-Amyloid deposits can directly cause neuronal damage in differentiated neurons of central nerves system (CNS), specifically in the AD brain. Hence, solubilizing and disaggregating β-amyloid plaques would be an effective approach towards AD treatment.

**Figure 1:**
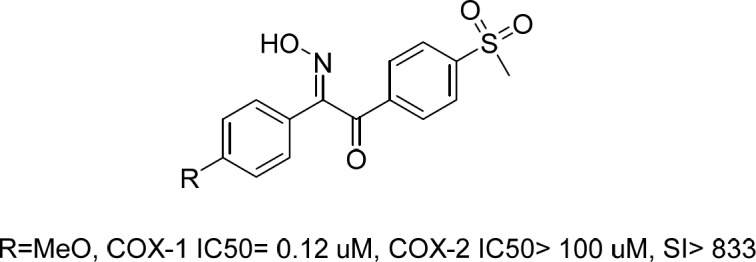
Previously reported compound as selective COX-1 and β-amyloid aggregation inhibitor

Recently, we reported a series of stillbenoid 2-hydroxyiminoethanone derivatives with sulfonyl methyl pharmacophore moiety as selective COX-1 and β-amyloid aggregation inhibitors (Figure 1) (16). Those compounds showed potent COX-1 inhibition activity and *in-vivo* anti-inflammatory potential. Correspondingly, their prominent structure and activity was further evaluated by comprehensive *in-silico* studies and X-ray crystallography to elucidate their unknown characteristics (17).

**Figure 2:**
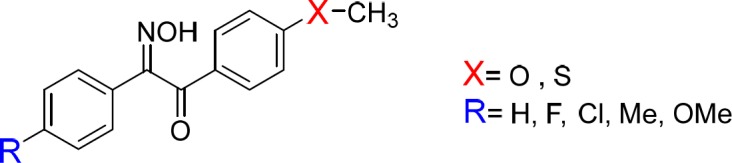
Structure of designed compounds as anti-inflammatory and β-amyloid aggregation inhibitors

These findings prompted us to synthesize and evaluate new derivatives of hydroxyiminoethanones (Figure 2) and evaluate them as *in-vivo* anti-inflammatory and β-amyloid aggregation inhibitors. To find out the role of sulfonylmethyl group as a strong electron-withdrawing moiety on the pharmacologic activity of compounds, it was replaced by strong electron-donating groups e.g. methoxy and methylthio in the new derivatives 1-10b. 

## Experimental

*Chemistry*

All starting materials, reagents, and solvents were purchased from Merck AG (Darmstadt, Germany) or Aldrich and used without further purification. Merck silica gel 60 F254 plates were applied for analytical thin layer chromatography (TLC) with detection by UV light (254 nm, UV lamp). Column chromatography was carried out on silica gel (230–400 mesh). Melting points were measured in open capillaries on a Stuart Scientific apparatus and are uncorrected. NMR spectra were recorded on a Bruker 400 or 500 MHz spectrometers using tetramethylsilane (TMS) as an internal standard, and chemical shifts (δ) and coupling constants (*J*) are expressed in ppm and Hertz (Hz), respectively. The IR spectra were recorded on a PerkinElmer FT-IR spectrophotometer (KBr disks). Low resolution mass spectra were measured using a HP 5975 Mass Selective Detector (Agilent technologies). Elemental analyses were carried out by ECS-4010 (Costech International S.p.A.) elemental analyzer. The results of elemental analyses (C, H, N) were within ± 0*.*4% of the calculated values.

*General procedure for the synthesis of compounds* ***1-10a***

Compounds were prepared according to our previously reported procedure (16, 18).

*General procedure for the synthesis of compounds* ***1-10b***

To a stirred solution of compounds **1-10a** (1 mmol) in methanol, sodium methoxide (1.2 mmol) was added and stirred for 5 min. Then, butyl nitrite (2 mmol) was added to the mixture and it was stirred for 30 min. After completion of the reaction, it was monitored by TLC, and then concentrated under vacuum. The product was then dissolved in water and adjusted to pH 7 by adding hydrochloric acid solution (2 M) and the aqueous phase was extracted with ethyl acetate (3 times). Then organic phase was dried and concentrated under vacuum. Column chromatography was performed to purify compounds on silica gel (230-400 mesh) eluting with methanol/chloroform (2%). All spectra data for compounds **1-10b** are in Supplementary material.

*2-hydroxyimino-1-(4-methoxyphenyl)-2-phenylethanone (* ***1b*** *)*

Yield 67%; mp: 130 °C; IR (KBr, cm^-1^) ν max: 3258.2 (O-H), 3067.8 (C-H_Ar_), 2937.7 (C-H_Aliph_), 1638 (C = O), 1595 (C = N). ^1^H-NMR (400 MHz, DMSO-d6) δ (ppm): 3.84 (s, 3H, OCH_3_), 7.11 (d, J=7.2 Hz, 2H, Ar-H), 7.31-7.33 (m, 3H, Ar-H), 7.38-7.40 (m, 2H, Ar-H), 7.37 (d, J = 6.8 Hz, 2H), 11.69 (s, 1H, NOH). ^13^C-NMR (125 MHz, DMSO-d6) dδppm: 56.32, 115.29, 126.21, 128.17, 129.65, 130.49, 131.99, 132.44, 155.43, 164.86, 193.97. MS m/z (%): 255.1 (M+, 35), 152.1 (25), 135.2 (100), 103.1 (30), 77.1 (44). Anal Calcd for C_15_H_13_NO_3_: C, 70.58; H, 5.13; N, 5.49. Found: C, 70.63; H, 5.04; N, 5.41.

*2-(4-fluorophenyl)-2-hydroxyimino-1-(4-methoxyphenyl) ethanone (* ***2b*** *)*

Yield 65%; mp: 138 °C; IR (KBr, cm^-1^) ν max: 1643 (C = O). ^1^H-NMR (400 MHz, DMSO-d6) dδppm): 3.85 (s, 3H, OCH_3_), 7.10 (d, J = 8.96 Hz, 2H, Ar-H), 7.26 (t, J = 8.92 Hz, 2H, Ar-H), 7.51 (dd, J = 5.44 Hz, J = 8.98 Hz, 2H, Ar-H), 7.81 (d, J = 8.92 Hz, 2H, Ar-H), 11.71 (s, 1H, NOH). ^13^C-NMR (100 MHz, DMSO-d_6_) dδ ppm): 56.19, 115.17, 116.60(d, J = 21.91 Hz), 127.89, 128.35 (d, J = 8.46 Hz), 131.88, 132.20 (d, J = 8.54 Hz), 154.27, 163.40 (d, J = 246 Hz), 164.79, 193.56. MS m/z (%): 273.0 (9), 152.0 (25), 136.0 (31), 135.0 (100), 122.0 (26), 121.0 (48), 107.0 (17) Anal. Calcd for C_15_H_12_FNO_3_: C, 65.93; H, 4.43; N, 5.13. Found: C, 65.88; H, 4.44; N, 5.16.

*2-(4-chlorophenyl)-2-hydroxyimino-1-(4-methoxyphenyl) ethanone (* ***3b*** *)*

Yield 52%; mp: 133 °C; IR (KBr, cm^-1^) vmax: 3365.2 (O-H), 3016.0 (C-H_Ar_), 2937.5 (C-H_Aliph_), 1667.2 (C = O), 1594.1 (C = N). 1H-NMR (500 MHz, DMSO-d6) dδ ppm): 3.75 (s, 3H, OCH_3_), 7.02 (d, J= 8.5 Hz, 2H, Ar-H), 7.39 (s, 4H, Ar-H), 7.72 (d, J = 9. 7 Hz, 2H, Ar-H), 11.76 (s, 1H, NOH). ^13^C-NMR (125 MHz, DMSO-d_6_) dδδ ppm): 56.35, 115.34, 127.92, 129.79, 131.29, 131.78, 132.06, 135.17, 154.41, 164.99, 193.56. MS m/z (%): 289.1 (M+, 5), 152.0 (22), 139.0 (28), 137.0 (56), 135.0 (100), 102.0 (21). Anal Calcd for C_15_H_12_ClNO_3_: C, 62.19; H, 4.18; N, 4.83. Found: C, 62.20; H, 4.13; N, 4.79.

*2-hydroxyimino-1-(4-methoxyphenyl)-2-p-tolylethanone (* ***4b*** *)*

Yield 54%; mp: 135 °C; IR (KBr, cm^-1^) ν max: 3409.1 (O-H), 3015.5 (C-H_Ar_), 2975.6 (C-H_Aliph_), 1667 (C = O), 1595.6 (C = N). ^1^H-NMR (400 MHz, DMSO-d6) dδ ppm): 2.29 (s, 3H, Ar-CH_3_), 3.84 (s, 3H, OCH_3_), 7.09 (d, J = 8.88 Hz, 2H, Ar-H) ,7.21 (d, J = 8.12 Hz, 2H, Ar-H), 7.36 (d, J = 8. 2 Hz, 2H, Ar-H), 7.8 (d, J = 8.84 Hz, 2H, Ar-H), 11.57 (s, 1H, NOH). ^13^C-NMR (100 MHz, DMSO-d6) dδ(ppm): 21.33, 56.16, 115.09, 125.98, 128.08, 129.57, 130.03, 131.78, 140.07, 155.23, 164.64, 193.89 MS m/z (%): 269.0 (M+, 12), 152.0 (32), 135.0 (100), 117.0 (46), 107 (17), 92.0 (27). Anal Calcd for C_16_H_15_NO_3_: C, 71.36; H, 5.61; N, 5.20. Found: C, 71.32; H, 5.58; N, 5.18.

*2-(4-methoxyphenyl)-2-hydroxyimino-1-(4-methoxyphenyl) ethanone (* ***5b*** *)*

Yield 49%; mp: 135 °C; IR (KBr, cm^-1^) ν max: 3324.6 (O-H), 3020.1 (C-H_Ar_), 2965 (C-H_Aliph_), 1650 (C=O), 1590 (C = N). ^1^H-NMR (500 MHz, DMSO-d6) dδ(ppm): 3.67 (s, 3H, OCH_3_), 3.75 (s, 3H, OCH_3_), 6.88 (d, J = 9 Hz, 2H, Ar-H), 7.01 (d, J= 9 Hz, 2H, Ar-H), 7.32 (d, J= 8.5 Hz, 2H, Ar-H), 7.71 (d, J = 8.5 Hz, 2H, Ar-H), 11.35 (s, 1H, NOH). ^13^C-NMR (125 MHz, DMSO-d6) δ (ppm): 55.87, 56.31, 115.11, 115.23, 124.84, 127.73, 128.21, 131.96, 155.11, 161.10, 164.79, 194.18. MS m/z (%): 285.0 (M+, 6), 207.0 (30), 152.0 (35), 135.0 (100), 133.0 (82), 103.0 (32), 90.0 (39). Anal Calcd for C_16_H_15_NO_4_: C, 67.36; H, 5.30; N, 4.91. Found: C, 67.32; H, 5.27; N, 4.93.

*2-(hydroxyimino)-1-(4-(methylthio)phenyl)-2-phenylethanone (* ***6b*** *)*

Yield 38%; mp: 138 °C; IR (KBr, cm^-1^) ν max: 1585 (C = N), 1655 (C = O), 2920 (C=CH), 3344 (C = OH). ^1^H-NMR (500 MHz, CDCl_3_) dδ(ppm): 2.50 (s, 3H, SCH_3_),7.28 (d, J = 8.5 Hz, Ar-H), 7.34 (t, J = 7.5 Hz, 2H, Ar-H), 7.39 (d, J = 6.5Hz, 1H, Ar-H), 7.53 (d, J = 7.5 Hz, 2H, Ar-H), 7.87 (d, J= 8.5 Hz, 2H, Ar-H). ^13^C-NMR (125 MHz, CDCl_3_) dδ(ppm): 14.55, 125.11, 126.38, 128.86, 129.71, 130.49, 130.86, 130.91, 148.31, 157.06, 192.93. Mass (M/Z, %): 259.1(3), 212.1(5), 183.1(5), 151.2(100), 123.1(20). Anal Calcd for C_15_H_13_NO_2_S: C, 66.40; H, 4.83; N, 5.16. Found: C, 66.44; H, 4.87; N, 5.13.

**Figure 3:**
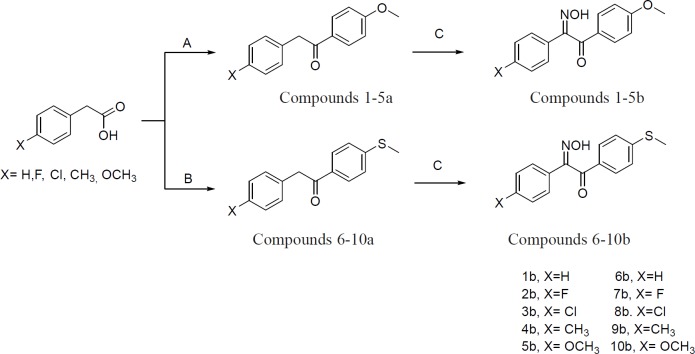
Synthetic route for compounds **1-10b**. A: (TFAA, H_3_PO_4_, Anisole, rt, stirr), B: (TFAA, H_3_PO_4_, Thioanisole, rt, stirr), C: (CH_3_OH, NaOCH_3_, BuONO, rt, stirr)

**Figure 4:**
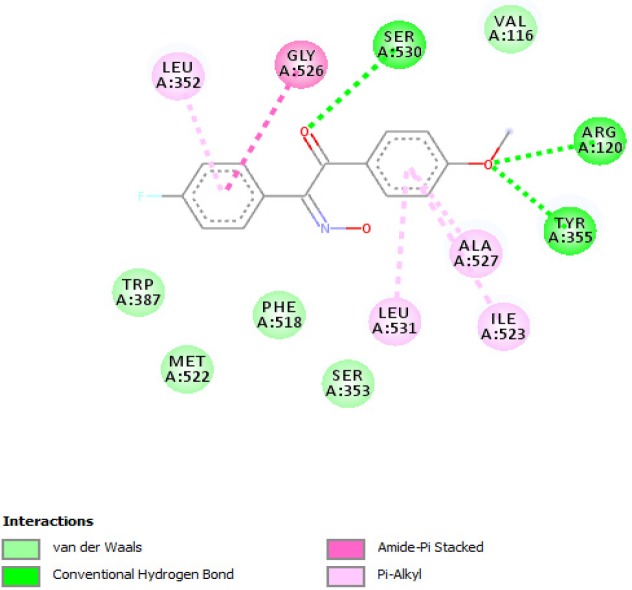
Binding conformation of **2b** and interacting amino acids in the active site of cyclooxygenase-1

**Figure 5:**
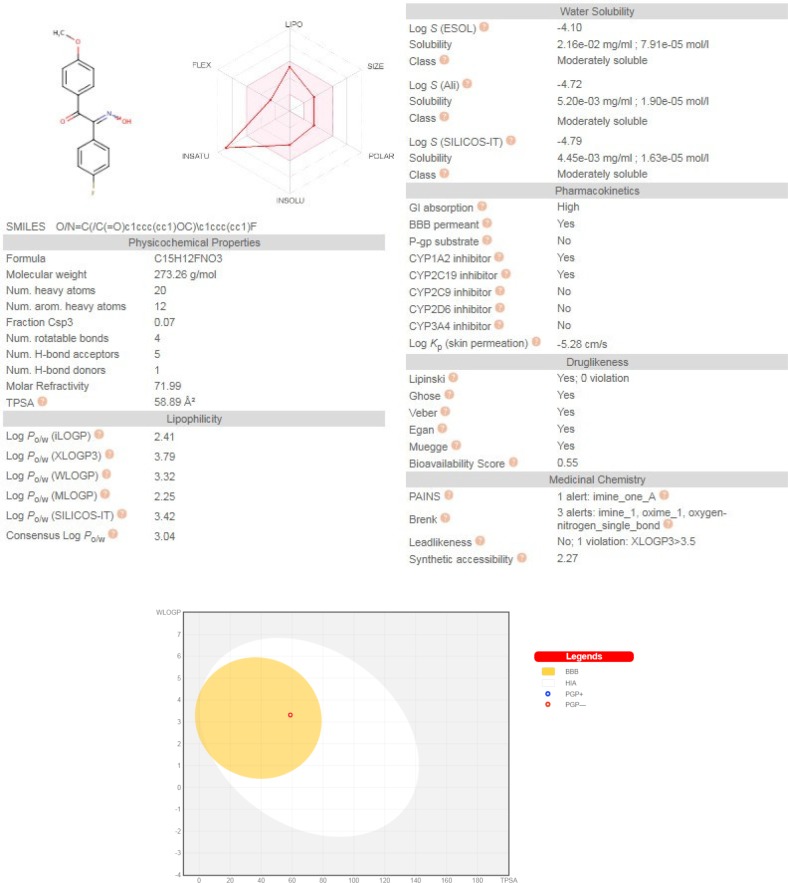
(a). Predicted physicochemical descriptors and pharmacokinetic parameters of compound **2b **by SwissADME web server. (b) BOILED-Egg chart relating two descriptors WlogP and TPSA for prediction of BBB permeability, human intestinal absorbtion (HIA) and P-gp substrate probability

**Table 1:** Percentage of β-amyloid aggregation after 48 h of treating by each compound. Significant values are indicated by asterisk

**Compound**	**Mean (%) ± SEM**
1b	61.96 ± 6.9 ***
2b	50.92 ± 2.2 ***
3b	52.41 ± 1.2 ***
4b	59.00 ± 6.6 ***
5b	51.91 ± 0.0 ***
6b	97.87 ± 2.0
7b	98.71 ± 5.3
8b	72.64 ± 7.2 **
9b	86.18 ± 6.0
10b	98.84 ± 4.3
Donepezil	71.20 ± 1.6 **
Rifampicin	67.02 ± 1.5 ***
Solvent control	100 ± 2.5

**, *p *˂ 0.001 compared with the control group.

***, *p *˂ 0.0001 compared with the control group.

**Table 2:** Effect of pretreatment of rats with Compounds **1 – 10b**, celecoxib or indomethacin on the carrageenan-induced paw edema and formalin test

**Compound**	**Change in paw volume** **a ** **(cm** **3** **) Mean ± SEM**	**AUC of pain score** **b ** **Mean ± SEM**
Control (DMSO)	0.37 ± 0.02	86.46 ± 4.79
1b	0.21 ± 0.06 *	72.08 ± 5.23
2b	0.39 ± 0.02	48.75 ± 9.95 *
3b	0.26 ± 0.01 *	62.04 ± 12.95
4b	0.30 ± 0.05	83.28 ± 14.76
5b	0.34 ± 0.06	57.03 ± 12.39
6b	0.21 ± 0.05 *	61.70 ± 4.26
7b	0.29 ± 0.03	72.00 ± 12.65
8b	0.48 ± 0.04	96.10 ± 7.40
9b	0.29 ± 0.04	76.38 ± 11.03
10b	0.46 ± 0.06	86.73 ± 8.73
Celecoxib	0.05 ± 0.02 ***	62.61 ± 4.71
Indomethacin	0.22 ± 0.05 *	ND

aPaw volume was measured before and 4 h after carrageenan injection into rat hind paw and the change in paw volume was calculated for each rat. Data were shown as mean ± SEM (N = 6-8 in each group).

b AUC of pain scores during 60 min of observation in formalin test. Data were shown as mean ± SEM (N = 6-8 in each group).

*p < 0.05 significant difference compared with the control group. ND Not determined.

*2-(4-fluorophenyl)-2-hydroxyimino-1-(4-(methylthio) phenyl)ethanone (* ***7b*** *)*

Yield 40%; mp: 138-140 °C; IR (KBr, cm^-1^) ν max: 1584 (C=N), 1656 (C=O), 2925 (C-H), 3353 (O-H). ^1^H-NMR (500 MHz, CDCl_3_) dδ(ppm): 2.51 (s, 3H, SCH_3_), 7.03 (t, J = 9 Hz, 2H, Ar-H) ,7.28 (d , J = 9 Hz, 2H, Ar-H), 7.52 (dd, J = 5 Hz, J = 9 Hz, 2H, Ar-H), 7.85 (d, J = 8.5Hz, 2H, Ar-H). ^13^C-NMR (125 MHz, CDCl_3_) dδ(ppm): 14.58, 116.08 (d, J = 21.25 Hz), 125.12, 127.3 (d, J = 2.5 Hz), 128.43 (d, J = 7.5 Hz), 129.71, 130.67, 148.60, 156.04, 163.98 (d, J = 250 Hz), 192.83. Mass (M/Z, %): 275.1 (2), 228.1 (4), 151.2 (100), 123.1 (17), 101.1(3), 79.2 (15). Anal. Calcd for C_15_H_12_FNO_2_S: C, 62.27; H, 4.18; N, 4.84. Found: C, 62.24; H, 4.15; N, 4.80.

*2-(4-chlorophenyl)-2-hydroxyimino-1-(4-(methylthio) phenyl)ethanone (* ***8b*** *)*

Yield 33%; mp: 120-122 °C; IR (KBr, cm^-1^) ν max: 1584 (C = N), 1661 (C = O), 3366 (O-H). ^1^H-NMR (500 MHz, CDCl_3_) d (ppm): 2.5 (s, 3H, SCH_3_), 7.28 (d, J = 9 Hz, 2H, Ar-H) ,7.31 (d, J = 9 Hz, 2H, Ar-H), 7.47 (d, J = 8.5 Hz, 2H, Ar-H), 7.83 (d, J = 9 Hz, 2H, Ar-H). ^13^C-NMR (125 MHz, CDCl_3_) d (ppm): 14.53, 125.11, 127.61, 129.13, 129.67, 130.65, 130.90, 136.56, 148.64, 156.07, 192.63. Mass (M/Z, %): 301.0 (5), 271.0 (4), 223.9(3), 168.0 (33), 151.0 (60), 133.0 (100), 108.0 (10), 90.0 (45). Anal Calcd for C_15_H_12_ClNO_2_S: C, 58.92; H, 3.96; N, 4.58. Found: C, 58.88; H, 3.93; N, 4.51.

*2-hydroxyimino-1-(4-(methylthio) phenyl)-2-p-tolylethanone (* ***9b*** *)*

Yield 18%; mp: 145-146 °C; IR (KBr, cm^-1^) ν max: 1584 (C = N), 1652 (C = O), 2952 (C-H_Aliph_), 3327 (O-H). ^1^H-NMR (500 MHz, CDCl3) dδ(ppm): 2.32 (s, 3H, Ar-CH_3_), 2.49 (s, 3H, SCH_3_), 7.13 (d, J = 8.5 Hz, 2H, Ar-H) ,7.27 (d, J = 9 Hz, 2H, Ar-H), 7.41 (d, J = 8 Hz, 2H, Ar-H), 7.86 (d, J = 8.5 Hz, 2H, Ar-H). ^13^C-NMR (125 MHz, CDCl3) dδ(ppm): 14.57, 21.40, 125.09, 126.32, 128.00, 129.59, 129.71, 130.86, 140.91, 148.22, 156.97, 193.06. Mass (M/Z%): 285.1 (M+, 40), 255.2 (5), 208.2 (8), 168.1 (7), 151.2 (100), 123.1 (15). Anal Calcd for C_16_H_15_NO_2_S: C, 67.34; H, 5.30; N, 4.91. Found: C, 67.29; H, 5.26; N, 4.94.

*2-(hydroxyimino)-2-(4-methoxyphenyl)-1-(4-(methylthio) phenyl)ethanone (* ***10b*** *)*

Yield 39%; mp: 127-128 °C; IR (KBr, cm^-1^) ν max: 1584(C = N), 1635(C = O), 2960(C-H), 3292(O-H). ^1^H-NMR (500 MHz, CDCl_3_) dδ(ppm): 2.50 (s, 3H, S-CH_3_), 3.83 (s, 3H, OCH_3_), 6.92 (d, J = 9 Hz, 2H, Ar-H), 7.24 (d, J = 9 Hz, 2H, Ar-H), 7.66 (d, J = 9 Hz, 2H, Ar-H), 7.91 (d, J = 8.5 Hz, 2H, Ar-H). ^13^C-NMR (125 MHz, CDCl3) dδ(ppm): 14.61, 55.29, 113.68, 124.67, 128.01, 130.77, 131.61, 132.42, 147.14, 154.65, 160.76, 190.54. Mass (M/Z, %): 271.1 (15), 168.0 (18), 151.0 (100), 136.9 (10), 122.9 (12), 103.0 (80), 76.0(40). Anal Calcd for C_16_H_15_NO_3_S: C, 63.77; H, 5.02; N, 4.65. Found: C, 63.72; H, 5.06; N, 4.62.

*Animals and drug administration*

Male wistar rats (200-250 g; Pasteur Institute, Tehran, Iran) were used in this study. The animals were housed on a 12 h light/dark cycle (12 h/12 h, lights on at 0700 h), controlled temperature (22 ± 2 °C), and humidity (30-40%) and with free access to standard food and tap water. Animal experiments were carried out in accordance with the Iranian Ministry of Health and Medical Education guidelines for care and use of laboratory animals and also were approved by the university Ethics Committee. 

The test drug or celecoxib were dissolved in dimethyl sulfoxide (DMSO) and were administered by intraperitoneal (i.p.) injection in a volume of 0.5 mL/kg 30 min before behavioral tests. The control group received DMSO (0.5 mL/kg) by i.p. injection. All tested and standard drugs were administered at 40 mg/kg.

*Formalin test*

Peripheral pain was induced in rats by intraplantar injection of formalin (0.04 mL, 5%) into the right hind paw. Pain-related behavior characterized by Dubuisson and Dennis (19), was recorded for 60 min after formalin injection as 0 = normal weight bearing on the injected paw, 1 = limping during locomotion or resting the paw lightly on the floor, 2 = elevation of the injected paw, and 3 = licking or biting the injected paw. Rats’ behaviors were continuously scored every 15 s and the area-under-the-curve (AUC) of the pain scores was calculated to show the overall pain score of each animal during the test.

*Carrageenan-induced paw edema*

Edema was induced on rat′s right hind paw by subcutaneous plantar injection of 0.1 mL of 1% carrageenan in saline. The test compounds and reference drugs were given 30 min before the injection of carrageenan. The volume of the right paw was measured using a method described by Fereidoni *et al.* (20) immediately before injection and 4 h after induction of inflammation. The results were obtained by calculating the volume difference before and after injection of the right paw 

(21).

*Inhibition of Aβ*_1–42_ *aggregation*

Inhibitory properties of compounds on amyloid β protein 1-42 aggregation was determined using a thioflavin T (ThT)-based fluorescence assay. Commercially available Aβ_1–42 _protein fragment (A9810, Sigma–Aldrich) was dissolved in phosphate-buffered saline (PBS) pH 7.4. 

50 µM Aβ_1-42 _was incubated at 37 °C for 72 h to induce peptide aggregation. 100 μM inhibitor and 5 μM Aβ_1–42 _were incubated at 37 °C for 48 h. The Aβ_1–42 _± inhibitor mixture was added to thioflavin T (ThT; 200 μM) in 50 mM glycine-NaOH buffer pH 8.0 and ThT excitation/emission was measured at 448 nm/490 nm using a SpectraMax® Microplate Reader. Rifampicin (100 μM, Sigma R-3501) and Donepezil (100 μM, Sigma D-6821) were tested as reference compounds. Aβ_1–42 _aggregation percents were determined by the following calculation: [(IFi/IFo)×100] where IFi and IFo are the fluorescence intensities obtained for Aβ_1-42 _in the presence and in the absence of inhibitors (22).

*Molecular docking*

Docking was performed by Autodock 4.2 and PDB code of 1Q4G was used for docking of compound **2b** in the active site of cyclooxygenase-1. Validation of our docking method was performed by redocking of co-crystallized ligand alpha-methyl-4-biphenylacetic acid into the active site of COX-1. The full details of our docking protocol and parameters are described in our previous article (17). 

*Statistical analysis*

Statistical analysis was performed using the GraphPad Prism software (La Jolla, California, CA) using one-way analysis of variance followed by the Dunnett’s test, and *p*-values less than 0.05 were considered statistically significant.

## Results and Discussion

*Chemistry*

Initially, phenylacetic acid derivatives in the presence of trifluoroacetic anhydride (TFAA), phosphoric acid, and anisole or thioanisole were converted to 1, 2-diarylethanone derivatives (**1-10a**). In the next step, 1, 2-diarylethanones **1-10a**, were treated with sodium methoxide and then butyl nitrite to produce 2-hydroxyiminoethanones **1-10b**. All chemical steps for preparation of compounds **1-10b** along with a brief reaction data are illustrated in Figure 3.

*β-Amyloid aggregation test*

To investigate the anti-Alzheimer′s potential of compounds **1-10b**, the ability of synthesized compounds to inhibit the aggregation of β-amyloid peptides was investigated using thioflavin T (ThT)-based fluorescence assay. Rifampicin and donepezil were used as reference drugs in this study. The results are shown in Table 1 as a percentage of β-amyloid (1-42) peptide aggregation in 48 h in the presence of drugs or compounds **1-10b** at 100 µM concentration. 

The results showed that the percentage of β-amyloid (1-42) peptide aggregation during 48 h in the presence of donepezil and rifampicin was 71.2% and 67.0%, respectively. The ability of compounds **1-10b** to reduce the aggregation of β-amyloid (1-42) peptide was varied from 50.9 to 98.8%. As shown in Table 1, most of the tested compounds have a significant anti-aggregation effect on β-amyloid (1-42) peptides, but the anti-aggregation effect of **1-5b** derivatives is significantly stronger than **6-10b**. Meaningfully, the results demonstrated that the anti-aggregation effect of **1-5b** is also better than the standard drugs rifampicin and donepezil. Interestingly, compounds **2b**, **5b,** and **3b** are respectively the best compounds to inhibit the aggregation of β-amyloid peptide with 50.9, 51.9, and 52.4 percent of amyloid aggregation. Therefore, compound **2b** was shown to be the most potent compound for disaggregation of amyloid peptides.

*In-vivo anti-inflammatory evaluation*

This study was undertaken to evaluate the anti-inflammatory and anti-nociceptive effects of **1-10b** derivatives on two classical models: Carrageenan-induced paw edema and formalin test. Initially, synthesized compounds **1-10b** were evaluated for their potential anti-inflammatory activity in mouse hind paw edema. Celecoxib and indomethacin were used as reference drugs in both studies. The results of this assessment are shown in Table 2. 

The anti-inflammatory effects of **1-10b** compounds were measured 4 h after the carrageenan injection, and the results were reported as change of hind paw volume. As seen in Table 2, compounds **1b**, **3b,** and **6b** could reduce the paw volume after carrageenan injection significantly compared to the control group (DMSO) (*p*-value ˂ 0.05). Correspondingly, reference drugs indomethacin and celecoxib were significantly more active than the control group in this test. Additionally, formalin test was also carried out in order to evaluate the anti-nociceptive effect of compounds **1-10b** and the results are shown in Table 2. Interestingly, only compound **2b** was the most effective one to significantly reduce the pain related behavior in animals treated with formalin. On the other hand, celecoxib as a reference drug did not show any reduction in peripheral pain induced by formalin.

*In silico calculations of binding conformation and ADME properties*

According to our previously published study, 2-hydroxyiminoethanones were reported to be selective cyclooxygenase-1 inhibitors by *in-vitro* cyclooxygenase inhibition assay (16). Recently, we reported a comprehensive *in-silico* study by conformational analysis and MM-PBSA calculations in which selectivity of stilbenoids for cyclooxygenase isoenzymes could be predicted (17). In the same study, the geometry of imine bond in 2-hydroxyiminoethanones was determined by X-ray crystallography and attributed to (E)-isomer. On the other hand, conformational analysis by DFT calculations and also X-ray crystallography revealed that the most stable conformation of diaryl-2-hydroxyiminoethanones is in the form of transoid. According to these data, molecular docking as a useful tool was used to rationalize the *in-vivo* anti-inflammatory activity of compounds **1-10b** towards cyclooxygenase-1 enzyme. In this manner, compound **2b** was selected as a representative for docking into the putative active site of cyclooxygenase-1 and the interacting residues are illustrated in 

Figure 4. As shown, the binding conformation of **2b** in the active site is in the transoid form and as discussed in our previous publication (17), flexible stilbenoid COX-1 inhibitors adopt transoid conformation in the binding site when inhibiting cyclooxygenase-1 enzyme and cisoid form in the active site of cyclooxygenase-2. Figure 4 shows that carbonyl oxygen atom of **2b** interacts with hydroxyl group of Ser530 by hydrogen bond and oxygen atom of methoxy group has hydrogen bonding with guanidine and hydroxyl group of Arg120 and Tyr355, respectively. Other hydrophobic interactions such as pi-pi or pi-alkyl are formed between non-polar residues Leu352, Leu531, Ile523, Gly526, and Ala527.

Freely accessible web-server SwissADME (http://swissadme.ch/index.php) (23) with a view to inquire pharmacokinetic aspects of the representative compound **2b**, was used and the results are illustrated in Figure 5. Predictions showed that **2b** has no violations of druglikeness rules such as Lipinski’s rule of five, Veber, Ghose, Egan, and Muegge. This compound has high GI absorption and no P-gp substrate probability which offers a good oral bioavailability. Interestingly, **2b** has BBB permeability which is a requisite property of CNS active drugs on neurodegenerative diseases.

The BOILED-Egg chart relating the two descriptor WLOGP and TPSA, basically supports the idea of its BBB permeability and high GI absorption of compound **2b **due to its TPSA of 58.89 and WLOGP of 3.32. Moreover, this chart showed that compound **2b **is not a substrate for P-glycoprotein, hence suggesting that its BBB uptake would not be hindered by P-gp efflux (24).

## Conclusion

In summary, a series of diaryl-2-hydroxyiminoethanones **1-10b** were synthesized and evaluated as potential anti-inflammatory and β-amyloid aggregation inhibitors. Among compounds, **1-5b** showed more potency than **6-10b** and also reference drugs donepezil and rifampicin to disaggregate β-amyloid precipitates. Compound **2b** was the best one and could reduce the extent of amyloid aggregation to 50.9%. Correspondingly, anti-inflammatory evaluation of these compounds by carrageenan and formalin tests revealed that compounds **1-3b** are capable of reducing inflammation and pain feeling significantly. 
